# Aircraft Noise and Quality of Life around Frankfurt Airport

**DOI:** 10.3390/ijerph7093382

**Published:** 2010-08-31

**Authors:** Dirk Schreckenberg, Markus Meis, Cara Kahl, Christin Peschel, Thomas Eikmann

**Affiliations:** 1 ZEUS GmbH, Sennbrink 46, 58093 Hagen, Germany; E-Mail: peschel@zeusgmbh.de (C.P.); 2 Hörzentrum Oldenburg GmbH, Marie-Curie-Str. 2, 26129 Oldenburg, Germany; E-Mail: M.Meis@Hoerzentrum-Oldenburg.de (M.M.); 3 Department of Psychology, University Hamburg, Von-Melle-Park 5, 20146 Hamburg, Germany; E-Mail: cara.kahl@uni-hamburg.de (C.K.); 4 Institute of Hygiene and Environmental Medicine, Faculty of Medicine, Justus-Liebig-University Giessen, Friedrichstr. 16, D-35392 Giessen, Germany; E-Mail: thomas.eikmann@hygiene.med.uni-giessen.de (T.E.)

**Keywords:** aircraft noise, annoyance, disturbance, non-acoustical factors, noise sensitivity, environment, health, quality of life, stress theory, HQoL, EQoL

## Abstract

In a survey of 2,312 residents living near Frankfurt Airport aircraft noise annoyance and disturbances as well as environmental (EQoL) and health-related quality of life (HQoL) were assessed and compared with data on exposure due to aircraft, road traffic, and railway noise. Results indicate higher noise annoyance than predicted from general exposure-response curves. Beside aircraft sound levels source-related attitudes were associated with reactions to aircraft noise. Furthermore, aircraft noise affected EQoL in general, although to a much smaller extent. HQoL was associated with aircraft noise annoyance, noise sensitivity and partly with aircraft noise exposure, in particular in the subgroup of multimorbid residents. The results suggest a recursive relationship between noise and health, yet this cannot be tested in cross-sectional studies. Longitudinal studies would be recommendable to get more insight in the causal paths underlying the noise-health relationship.

## 1. Introduction

Frankfurt Airport (Frankfurt am Main, Germany) is an important international airport in Europe with an estimated 486,000 movements (10% at night-time), 53 million passengers and 2 million tons of cargo (in 2008). For 2020 about 701,000 movements (88 Mio passengers, more than 3 million tons of cargo) are predicted. In order to manage this predicted amount of movements it is intended to construct a new 4th runway to increase the current capacity of 83 to 120 flight movements per hour. The opening of the new runway is expected in 2011.

After the announcement of the airport expansion in 1998 a regional mediation process started and a round table, the Regional Dialogue Forum Frankfurt Airport (RDF), was formed in order to continue information on and discussion about the development of the airport. Members of the RDF are representatives of action groups, local authorities, trade unions, churches, regional industry, and aviation industry. After a feasibility study about the assessment of aircraft noise effects was carried out in 2003 [1] the RDF commissioned a main field study on the effects of aircraft noise in communities in the vicinity of Frankfurt Airport. This main field study (FRA-S) was carried out from 2004 to 2006 and took place before the final approval decision about the expansion was made at the end of 2007. The study aimed at assessing the reactions to aircraft noise of residents around an international airport in a situation between the announcement and the planned implementation of the expansion of the airport. The objectives of the field study in particular were:

▪ to assess the impact of aircraft noise before airport expansion, *i.e.*, the construction of the new 4th runway;
▪ to get an update of the regional exposure-response relationship for aircraft noise annoyance and disturbances due to aircraft noise (communication, restoration, concentration/work, sleep);
▪ to get information about the *status quo* of environmental and health-related quality of life and any effects of aircraft noise on that *status quo*.

A report with the results of the study was finalized in 2006 [2]. This article presents the main findings of FRA-S with regard to reactions to aircraft noise (annoyance) and more comprehensive outcomes concerning the environmental and health-related quality of life.

## 2. Working Model of Aircraft Noise Effects

To meet the objectives as defined by the RDF the study comprises, beside the assessment of aircraft noise exposure, instruments for the ascertainment of aircraft noise annoyance and its non-acoustical co-determinants, as well as instruments for the assessment of environmental (EQoL) and health-related quality of life (HQoL).

The underlying theoretical concept used as a working model in this study is based on noise-related stress models [3,4] referring to the transactional stress concept of Lazarus and colleagues [5]. These models describe the relationship between noise exposure, coping, and annoyance [4], and further mental as well as physical health outcomes [3]. That is, long-term noise annoyance can be understood as strain (reappraisal) resulting from an assessment process including the perceived disturbance and annoyance due to the sound (primary appraisal) and the perceived control over the noise situation [6], *i.e.*, among others the perceived possibilities to cope with noise [3] (secondary appraisal). Chronic psychological strain, going along with physiological stress reactions to noise exposure [7] may increase the risk of health problems, in particular cardiovascular diseases [7] and/or disorders in mental health [8].

Whereas van Kamp [3] describes the role of appraisal of stressors (noise), activation, and coping with the noise for the prediction of health complaints, Stallen [4] points out the importance of the social aspect of noise (“you expose me”) on perceived control and, thus, on annoyance and further source-related attitudes. Stallen’s model provides a theoretical frame for the often found associations between non-acoustical, attitudinal factors (e.g., attitudes towards the source and towards authorities) and noise annoyance [9–11] indicating that these attitudes co-determine noise annoyance in a similar of even higher extend than the annoying sound itself [10,12]. Stallen identifies the noise policy or the way the sound production is managed as a second external stimulus of stress reactions to noise in addition to the sound itself. This social-psychological perspective of noise reactions is supported by findings about the impact of procedural (un-)fairness [13] and the regional political discourse [14] on aircraft noise annoyance.

In environmental psychological approaches the role of the perceived environmental context on human’s well-being and health (person-environment fit) has been emphasized for many years and stress models as described above are supplemented by the description of the restorative as well as aversive impact of the (physical) environment [15]. Following this research perspective, aircraft noise can be understood as an environmental stressor affecting the perceived environmental quality as well as stress-induced health outcomes.

In a similar way, the multi-dimensional concept of quality of life, including aspects of emotional, functional, mental, physical, and social well-being as perceived by the individual [16], offers a wide frame to investigate the possible health-related outcomes of (aircraft) noise. In several studies the association between transportation noise, environmental (EQoL) and health-related quality of life (HQoL) was investigated [17–19]. In this study, in line with the suggestion of Lercher [20] to combine transactional and contextual stress models (including environmental context factors) and to conceptually integrate the notion of EQoL and HQoL in environmental health impact assessment, the noise-related stress concept and the deduced instruments and assessments include the following aspects:

▪ Aircraft noise exposure as the environmental stressor of interest.
▪ Psychological reactions to aircraft noise: disturbances due to aircraft noise, measures to cope with aircraft noise and—as a key psychological stress reaction—aircraft noise annoyance, defined as “a psychological concept which describes a relation between an acoustic situation and a person who is forced by noise to do things he/she does not want to do, who cognitively and emotionally evaluates this situation and feels partly helpless” [21, p. 525].
▪ Contextual, personal and attitudinal (social) factors potentially co-determining noise reactions
▪ Sleep quality potentially affected directly by aircraft noise exposure at night or indirectly by the reactions to aircraft noise at daytime.
▪ Health-related variables as further outcomes of aircraft noise: health complaints, HQoL.
▪ EQoL: Residential satisfaction in total and with regard to infrastructure, quietness, attractiveness.

Note, that, although there is evidence of impacts of noise on health (mediated by psychological noise reactions), the aircraft noise exposure-annoyance-health association can also be interpreted the other way around: that is, vulnerable people—those who are more sensitive to noise and/or those who suffer from pre-existing illness—may have reduced behavioural or cognitive resources to cope with the noise exposure and therefore react with stronger annoyance to the noise and, hence, perceive a reduced HQoL [22]. It was shown in other publications concerning the FRA-S data that the prevalence of chronic and acute health diseases ever diagnosed by a doctor as well as the frequency of medicine use were not associated with aircraft noise exposure in terms of higher prevalence of diseases and medical consumption with increasing aircraft sound levels [23,24]. However, several diagnosed diseases and the use of headache drugs, sleeping drugs, calmatives, and asthma drugs were found to be associated with noise sensitivity [24], an individual disposition that, while independent from noise exposure, increases the susceptibility of an individual to noise in general [25]. Whether noise sensitivity and the diagnosed health diseases and medical consumption, respectively, are both indicators of a general ‘vulnerability’ [26,27] or of a common underlying personal dimension such as neuroticism [28] or negative affectivity [29,30], or whether pre-existing illnesses modify the sensitivity to noise (and other environmental stressors) in general, and therewith causes elevated reactions to noise, is not yet clear. Nevertheless, it is plausible to assume that most of the assessed diagnosed diseases and medical consumption indicate objective health problems and therewith resident’s morbidity which (pre-)exists independently from the aircraft noise exposure. It is further hypothesized that multimorbidity—here defined as the occurrence of two or more health diseases –, cause, similar to noise sensitivity, a reduced ability to cope with aircraft noise and in line with this moderates the impact of aircraft noise on HQoL.

Similar to the health variables, residential satisfaction and noise reactions such as annoyance may be reciprocally associated with each other. Several studies found associations of residential satisfaction with noise annoyance [31,32]. It is somewhat unclear whether residential satisfaction is a secondary reaction to noise (mediated by annoyance) or a modifier of noise reactions prior to noise annoyance or both.

**Figure 1.**
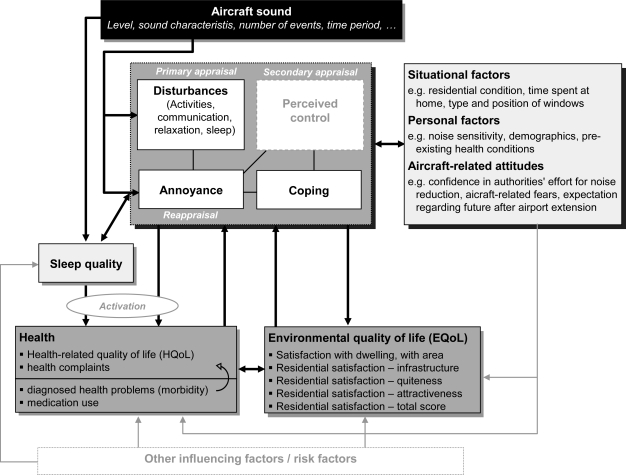
Conceptual working model of aircraft noise effects.

The different variables of reactions to noise, further outcomes with regard to HQoL and EQoL as well as potential personal, attitudinal and situational factors co-determining these variables are included in a summarized conceptual model of aircraft noise effects in Figure 1. It is not the aim of this paper to verify this model in detail. In FRA-S the working model was rather used as an orientation for the development of the questionnaires and the statistical analyses.

## 3. Methods

### 3.1. Sample and Procedure

The field study on the effects of aircraft noise on residents’ quality of life was carried out in 2005 in communities within a 40-kilometre distance from Frankfurt Airport. The subjects were sampled using a stratified random sampling method. That is, 66 residential areas were selected according to the aircraft noise exposure in 2003 with equivalent sound level contours for daytime L_Aeq,16h_ (6 *am* to 10 *pm*) as strata (see [2] for more details). Within the selected areas a total of 3,795 randomly selected residents was asked for an interview, of which 2,312 took part in the study (response rate 61%). The interviews were carried out from April to December 2005. The month in which a subject was contacted by the interviewer was selected at random. The participants were interviewed in face-to-face interviews (on average 45 minutes long) with regard to their residential situation, health-related quality of life, annoyance and disturbances due to noise, in particular to aircraft noise (study part I). The exposure to noise from aircraft, railway and road traffic noise was calculated for the address of each participant. In addition, a subsample of 200 persons assessed on four successive days their hourly aircraft noise annoyance, main activity, location, and—in case of indoor stay—the window position (study part II). This article presents the results of study part I.

### 3.2. Measures

#### 3.2.1. Noise Exposure

For the address of each participant aircraft noise exposure was modelled on the base of the flight movements of the six busiest months of the year 2005 according to the German aircraft noise calculation procedure with aircraft categories as proposed by the German Federal Environment Agency in 1999 (*AzB-99*; [33]). Several acoustical parameters were calculated including the equivalent sound level (L_Aeq_), maximum sound level (L_max_), and number of events (flight movements) above specified thresholds. For the analyses described in this article, aircraft noise load was indicated by the equivalent sound level for daytime (L_Aeq,16h_; 6 *am*–10 *pm*), night-time (L_Aeq,8h_; 10 *pm*–6 *am*), and for 24 hours of the day using the Day-Night level L_dn_ (including a penalty of 10 dB(A) for the night-time) as well as the Day-Evening-Night Level L_den_ (including a penalty of 5 dB(A) for the evening and 10 dB(A) penalty for the night-time). In addition, address-related road traffic and railway sound levels for daytime (L_Aeq,16h_) and for the night-time (L_Aeq,8h_) were assessed on the base of noise maps.

#### 3.2.2. Questionnaire

According to the conceptual model of aircraft noise effects described above, the following topics were assessed in the questionnaires:

▪ Residential situation and residential satisfaction
▪ Reactions to environmental noise, in particular aircraft noise
▪ Attitudes related to aircraft and Frankfurt Airport in general
▪ Health-related variables: health-related quality of life, health complaints, diagnosed diseases, use of medicine, sleep quality
▪ Personal factors: socio-demographic factors, individual noise sensitivity

The variables assessed in the questionnaire and analyzed within the context of this paper are listed in Appendix 1 of this article.

## 4. Results

Altogether, 2,312 residents were interviewed in the field study. In one case the address was not matched to the correct Gauss-Krueger coordinates, which were necessary to estimate address-related aircraft noise exposure. Therefore, the statistical analyses are based on data of 2,311 persons. The sample distributions of the study participants with regard to gender, age, socio-economical status, and aircraft noise exposure are shown in tables 2 and 3.

### 4.1. Aircraft Noise Annoyance

Results of correlation analyses between parameters of aircraft noise exposure and the aircraft noise annoyance experienced by the interviewed residents indicate that aircraft noise annoyance is associated with sound levels (equivalent, mean maximum sound level) as well as with the number of flyovers (N55, N70). However, the strongest exposure-annoyance relationship for aircraft noise was found between the equivalent sound level and aircraft noise annoyance (Table 4).

**Figure 2.**
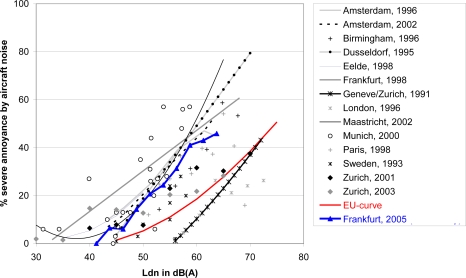
Dose-response data for severe aircraft noise annoyance from several surveys using a cut-off point of 70–75% of response scale for definition of high annoyance (HA). Source: van Kempen, und van Kamp ([42], p. 25, Figure 3b)—modified and supplemented; EU-curve: generalized dose-response curve for aircraft noise annoyance [43,44]. Source of the data of Zurich 2001/2003: Brink *et al.* [45]. Blue line and dots: data of the Frankfurt Noise Effect Study presented in this paper. References of all the other studies: see [42].

Exposure-response relationships were analyzed for the percentage of highly annoyed people. According to Schultz [41], a person has been defined as being highly annoyed (HA) when he or she chose the upper 27–28% of categories of the annoyance scale. This is the case for annoyance judgments of category 8 or higher on the 11-point scale. Results of this study with regard to the percentage of people highly annoyed by aircraft noise (%HA) was compared with findings of other international studies. Figure 2 shows the international comparison with regard to %HA related to the Day Night Level L_dn_ [42]. As can be seen, moderate sound levels already lead to severe noise annoyance due to aircraft noise. Compared to the generalized curve for %HA due to aircraft noise revealed by the meta-analysis of Miedema and Oudshoorn [43], also published in the EU position paper on noise annoyance with regard to L_den_ ([44] see red ‘EU-curve’), the blue ‘Frankfurt curve’ indicates higher annoyance at a given Day Night Level L_dn_. Nevertheless, the ‘Frankfurt 2005-curve’ is largely in line with most of the findings of the other field studies presented in Figure 2 and with results of further recently published studies not presented in Figure 2 [46,47]. The underlying data of the ‘EU-curve’ date from 1965 to 1992. Some authors suggest that the recently published studies on aircraft noise annoyance not included in the meta-analyses of Miedema and colleagues indicate a trend of increasing aircraft noise annoyance at a given sound level over the last decades [42,47,48]. These authors consider the respective EU-curve on aircraft noise annoyance as outdated.

In order to identify further aircraft noise reactions and non-acoustical factors associated with aircraft noise annoyance correlation analyses have been done between aircraft noise exposure, annoyance, and further reactions to aircraft noise as well as attitudinal, situational, and personal factors. The coefficients are presented in Table 5.

Aircraft noise annoyance is relative highly correlated with all disturbance judgments, both with disturbances of daily activities indoors (day and night) and outdoors (Table 5). In accordance with this result, with increasing sound levels and aircraft noise annoyance residents more often take measures to cope with the aircraft noise and to avoid disturbances due to aircraft noise. The results in Table 5 further indicate that the source-related attitudes and expectations are associated with aircraft noise annoyance. This is in line with results of many field studies on community reactions to noise [9,11]. These attitudes are also in a less degree but still significant (except positive expectations) correlated with the aircraft sound level. The correlation with aircraft noise exposure decreases after adjusting for annoyance. This indicates that the attitudes can be understood as (secondary) reactions to aircraft noise partly mediated by annoyance. This is confirmed by the finding that each partial correlation between aircraft sound level and annoyance controlled by each attitudinal factor is marginal lower in comparison to the zero-order correlation between aircraft sound level and annoyance. The interpretation of the source-related attitudes as secondary to aircraft noise annoyance is also supported by results of structural equation modeling done by Kroesen and colleagues, who found that none of the paths from the psychological factors to aircraft noise annoyance were significant, whereas for a part of the attitudinal factors (concern about negative health effects of noise, belief that noise can be prevented) the reverse path from the annoyance to the attitudes was statistically significant [49].

Among the personal factors the individual noise sensitivity is correlated with aircraft noise annoyance (r = 0.36) but as expected not with the aircraft sound level. In comparison to this socio-demographical factors play a minor role for aircraft noise annoyance as results of two-factorial ANOVAs with aircraft noise annoyance [11-point scale] as the dependent variable and 5-dB-L_den_-class as well as each of the selected grouped socio-demographic variables as independent factors suggest. This is in line with previous research [9]. However, some effects of these variables on annoyance were found, although with little effect size: Age was found to be non-linear related to aircraft noise annoyance, that is annoyance due to aircraft noise was higher in the group of middle-aged adults (40–60 years) in comparison to those younger or older than this group (F[2;2229] = 11.14, p < 0.001, ŋ_p_^2^ = 0.01). This non-linear effect of age on noise annoyance is also reported by Miedema and Vos [11] and van Gerven *et al.* [50].

Interviewed residents with a lower socio-economic status reported less annoyance due to aircraft noise than residents with middle and higher socio-economic status (F[1;2252] = 14.80, p < 0.001, ŋ_p_^2^ = 0.01). In accordance with this house owners were found to be more annoyed by aircraft noise than tenants (F[1;2252] = 60.77, p < 0.001, ŋ_p_^2^ = 0.03). Probably those residents who could afford ownership fear the loss of house values and in line with this are more annoyed by aircraft noise in comparison to those without properties. In fact, the fear of diminished house prices is correlated with aircraft noise annoyance (r = 0.54, p < 0.001) and with aircraft sound level L_den_ (r = 0.17, p < 0.001). As expected the correlation coefficients are much higher for house owners (house price–annoyance: r = 62, p < 0.001; house price–L_den_: r = 0.32, p < 0.001) than for tenants (house price–annoyance r = 0.37, p < 0.001; house price–L_den_ r = −0.09, p = 0.006).

### 4.2. Environmental Quality of Life

**Figure 3.**
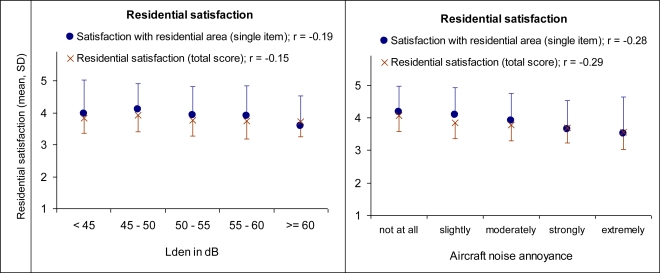
Means and standard deviation of residential satisfaction (single item, total score) by aircraft noise exposure (left side) and by aircraft noise annoyance (right side).

Table 5 shows that the residential satisfaction, in particular the satisfaction with the residential area outside the dwelling (single item and total residential satisfaction score including mainly area-related attributes), is correlated with annoyance and—weakly but significantly—with aircraft noise exposure. In particular satisfaction with house insulation and quietness in the residential area are both correlated with aircraft noise exposure and annoyance. In the partial correlation analyses between aircraft noise exposure and the satisfaction scores controlled by annoyance, the exposure-satisfaction association diminishes (except for satisfaction with house insulation and quietness) in comparison to the respective zero-order correlation. However, the annoyance—exposure correlation remains almost the same in the partial correlation analyses controlled by residential satisfaction. The correlation between satisfaction with quietness and aircraft noise exposure decreases somewhat after control for annoyance, but remains on a relative moderate level. This indicates that residential satisfaction, in particular the satisfaction with house insulation and quietness in the local area, can be interpreted as a secondary reaction to aircraft noise exposure partly mediated by annoyance. Note, that the aircraft noise exposure-annoyance correlation also decreases after control for the satisfaction with house insulation and quietness, suggesting that the annoyance may in turn partly be moderated by the satisfaction with house insulation and quietness. All in all, for residents living in the vicinity of Frankfurt Airport the results of the correlational analyses indicate that being stressed by aircraft noise lessen the satisfaction with the residential area and, thus, the perceived local environmental quality of life in general (see also Figure 3).

### 4.3 Health Related Quality Of Life (SF12/36), Sleep Quality, and Health Complaints

The following tables show descriptive statistics for the health complaints and SF12/36 scores as indicators of HQoL and for sleep quality (PSQI score) as indicator of nocturnal HQoL. The statistics are grouped by aircraft sound level for daytime and night-time (Table 6) and by aircraft noise annoyance and noise sensitivity (Table 7). Although on a descriptive level subjects of different sound level groups differ with regard to single health variables, no systematic increase with increasing noise exposure could be observed. Actually, HQoL with regard to vitality and mental health decreases with increasing aircraft sound level at daytime from <45 dB(A) up to the sound level class 50–55 dB(A), but then increases again for residents exposed to higher sound level classes at daytime. Similar, residents exposed to the lowest and highest aircraft sound level classes for daytime and night-time reported less health complaints with regard to the stomach, the limbs and in total than residents with aircraft noise exposure in between these sound level classes. The sleep quality is worst for residents exposed to 50 to 60 dB(A) at daytime and 50 to 55 dB(A) at night-time than for residents with less or higher aircraft noise exposure.

Accordingly, with increasing aircraft sound levels no increase in the risk (odds ratio) of HQoL below average, bad sleep quality and in the intensity of health complaints above average could be observed in logistic regression analyses with the health-related variables as criteria and aircraft noise exposure at daytime (for sleep quality: at night-time), annoyance, and noise sensitivity as predictors (Table 8). Similar results of the regression analyses were observed when the predictor L_Aeq_ for daytime was exchanged with L_Aeq_ for night-time. All regression analyses were adjusted for age, gender, socio-economical status, home ownership, residential satisfaction, usual window position in the sleeping room at night, and number of hours away from home. For analysing the impact of aircraft noise on physical health, e.g., cardiovascular risk effects in noisy areas it is obvious and a gold standard also to adjust regression models as described above for variables like body mass index, smoking and alcohol usage. But this study aimed at the effects of aircraft noise on annoyance, subjective health, environmental quality, and HQoL. For this purpose we decided in the study protocol in the beginning of the study not to include all these variables, due to budget limit and time limit of the duration of the interviews.

Table 8 shows that the health-related variables are proportionally related to psychological reactions to noise, indicated by annoyance due to aircraft noise. That is, the risk of health complaints (GSCL-24 scores), bad sleep quality (PSQI), and poor SF12/36 HQoL scores are related to annoyance indicating lower health-related quality of life with increasing aircraft noise annoyance. However, for the SF12 mental health score in the model including L_Aeq,16h_ as predictor this association failed the level of significance.

In addition, the risk of reduced HQoL is associated with an increase in individual sensitivity to noise with regard to all assessed HQoL variables. The results hold true for logistic regression analyses with sound level and annoyance as continuous as well as categorized predictors with the lowest class of sound level and annoyance as reference. The findings are similar for regression models including both sound level and annoyance as predictors and for separate models with either sound level or annoyance as predictor. Logistic regression models calculated separately for males and females reveal similar results.

Whether the “V”-shaped differences in HQoL across the aircraft sound level classes (see Table 6) persist in different subgroups distinguished with regard to socio-demographic, attitudinal (expectation concerning the future QoL after airport expansion), situational (usual window position), and personal (noise sensitivity, multi-morbidity) factors was tested in two types of GLM (with a significance level of p < 0.01). The first type of GLM includes aircraft sound level, age, gender, and socio-economical status as independent variables and selected HQoL variables (SF12/36 scores, total health complaints, sleep quality) as dependent variables. The second type includes, beside aircraft sound level, the attitudinal, situational, and personal factors as independent variables.

Due to limited space in this paper not all results of the GLM are presented here (see [2] for more details). To summarize: no interaction occurred that would indicate a significant moderating effect of the socio-demographic variables on the impact of aircraft-noise exposure on health outcomes. Significant main effects were observed with regard to sound level (see Table 6), age (older residents reported lower HQoL than younger), gender (female residents reported lower HQoL than males), and socio-economical status (residents with lower status reported lower HQoL than residents with higher status).

**Figure 4.**
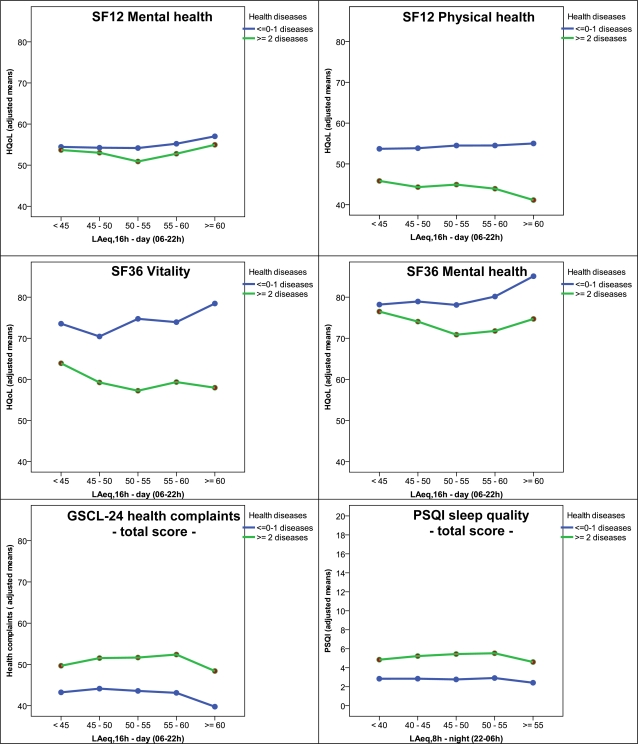
Results of GLM: Adjusted means of HQoL (SF12/36 scores, total health complaints, PSQI sleep quality) by aircraft sound level classes (L_Aeq,16h/8h_) and morbidity.

Results of the second type of GLM indicate higher HQoL for residents with up to one diagnosed health disease in comparison to those with two or more diseases, lower HQoL for those reporting negative expectations with regard to future (residential) life and for those judged themselves as being higher sensitive to noise in general compared to the lower noise-sensitive residents. With regard to potential impacts of aircraft noise on HQoL in subgroups of the residents the interactions between the described non-acoustical variables and aircraft noise exposure are of interest. Statistically significant interactions with aircraft sound levels were observed for the usual window position at daytime and for noise sensitivity. Yet, these interactions reflect marginal effects and cannot be interpreted in terms of a systematic moderating effect on the aircraft noise-HQoL relationship. This is somewhat different for the interaction morbidity x L_Aeq_ (for night-time concerning the criterion ‘PSQI sleep quality’ and for daytime with regard to the other HQoL criteria); see Table 9 and Figure 4. As can be seen from Figure 4, in the subgroups of residents reporting at least two health diseases (ever) diagnosed by a doctor, HQoL decreases somewhat with increasing aircraft noise exposure. This is particular true for residents exposed to lower to middle-ranged aircraft sound levels up to about 55 dB LAeq with regard to the SF12/36 scores (except SF12 mental health). In contrast to this, the HQoL of residents with less than two diseases remains constant or increases somewhat with increasing aircraft sound level. This interaction is not observed with regard to the reported health complaints and sleep quality. However, the described interaction confirms the notion of pre-existing health problems moderating the impact of (aircraft) noise exposure on health-related quality of life as described above in section 2.

One reason for the finding that above 50–55 dB(A) there is no consistent decrease in HQoL with increasing aircraft sound level could be a kind of self-selection, *i.e.*, people with severe health problems have moved away or decided not to live in high aircraft noise-exposed areas in the vicinity of Frankfurt Airport. But this *post hoc* explanation cannot be proved with the present data, because no information about migration is available in this study. However, length of residence was assessed in the questionnaire. Nonetheless, adding this variable as a covariate in the GLMs described above does not reveal more information or lead to alternative conclusions.

In a pilot study, Cischinsky *et al.* [51] investigated the in- and out-migration in the region around Frankfurt Airport (Rhine-Main region). Although aircraft noise was not the most important reason for the migration it became more important on subsequent motivation ranks. Nevertheless, because high aircraft noise-exposed areas in the vicinity of Frankfurt Airport have also other infrastructural disadvantages, a clear conclusion about the causal link between aircraft noise and migration motivation could not be drawn in the study of Cischinsky and colleagues.

The result of an association between (severe) aircraft noise annoyance and HQoL is confirmed by results from other studies [52,53]. Results of the adjusted regression analyses suggest furthermore an association independently from the annoyance between noise sensitivity and most of the investigated health variables. This is in line with other studies that report relations between noise sensitivity, annoyance, and health complaints [27,54,55]. Yet, the causal path of the association between noise annoyance, noise sensitivity, and health effects is not clear. There are mainly three different explanatory approaches and interpretations discussed with regard to this issue: (1) Noise sensitivity is an indicator of an individual’s vulnerability, which is closely related to (reported) health problems and which modifies individual noise reaction, suggesting that the noise exposure-annoyance-health relationship itself may be spurious [26]; (2) The noise sensitivity-annoyance-health relationship responsible for the dilution of a direct association between noise exposure and (reported) health incorporates a recall bias, which is absent when noise sensitivity is assessed before the occurrence or diagnosis of health disorders [56]; (3) The pre-existing health status and noise sensitivity are two interrelated ‘vulnerability’ factors which sap one’s energy to cope with noise (and other stressors), and, thus, moderate the impact of noise exposure on noise reactions (annoyance) as well as on HQoL in general [22,27,57].

It seems that ‘recall bias’ is not the whole story. This interpretation of the findings neglects the relationship between noise sensitivity and physiological functions [58,59]. And, a recall bias would be more plausible in terms of reported health complaints (misleadingly?) attributed to noise but not in terms of a positive noise sensitivity-health association diluting a direct noise-health association. The third explanation seems to be the most plausible one. It fits with results of previous noise-related studies about the effect of health status on noise reactions [22,27,60]. It is also in line with general stress models recognizing pre-existing chronic health problems as stress-enhancing [61] and partly with results of this study, where it was shown that among the multimorbid residents reported HQoL decreased somewhat with increasing aircraft sound levels at least in low to middle-ranged sound level classes.

## 5. Conclusions

In 2005 a field study about residents’ responses to aircraft noise was carried out in 66 residential areas in the vicinity of the Frankfurt International Airport. Residents (2,312) were interviewed with regard to their reactions to aircraft noise and their environmental as well as health-related quality of life. For the address of each participant sound levels for aircraft, road traffic, and railway noise were assessed. The study took place between the announcement (in 1998) and the approval decision (at the end of 2007) of the airport expansion (construction of a 4th runway).

Among several indicators of aircraft noise exposure the equivalent sound level showed the highest correlation with aircraft noise annoyance. The percentage of people (highly) annoyed by aircraft noise was found to be higher than predicted from general exposure-response curves. However, the degree of aircraft noise annoyance in communities around Frankfurt Airport is, all in all, in line with results from other recently published studies. Beside the sound level, non-acoustical factors, in particular the expectations with regard to future residential life after airport expansion and the confidence in authorities’ effort for aircraft noise reduction, were associated with the reactions to noise and with HQoL. The results of this study indicate that aircraft noise exposure not only has an impact on noise-specific (stress) reactions but also—although with much lower effect size—on perceived EQoL in general.

The HQoL variables were found to be associated with aircraft noise annoyance as well as with the individual noise sensitivity. The more residents were annoyed by aircraft noise the poorer was their HQoL. This is in particular true for higher noise-sensitive residents than for lower sensitive ones. In addition, within the group of multimorbid residents an association between aircraft sound level and HQoL was observed. However, again, this effect was rather small.

All in all, it could be shown that the impact of aircraft noise on residents living in the vicinity of an airport effects noise-specific stress reactions (annoyance, disturbances) as well as QoL in general. Yet, the strengths of the impact of aircraft noise exposure on QoL decreases coming from noise-specific reactions (e.g., annoyance) to environmental-specific reactions (EQoL) and finally to health-related outcomes (HQoL). Furthermore, it became obvious that the noise-HQoL relationship is not a simple, uni-directional one. It is likely that aircraft noise affects the health of people in particular when they face limited resources to cope with the noise, e.g., due to pre-existing illness and/or elevated sensibility to noise in general. Limited coping ability, again, enhances the strain and enables the development of further stress-related health problems and limitations in HQoL. Admittedly, this assumed recursive process cannot be tested in cross-sectional studies, nor in experimental studies on acute noise reactions. Longitudinal studies would be recommendable to get more insight in the causal paths underlying the noise-health relationship.

## Figures and Tables

**Table 2.** Number of participants by gender, age, and socio-economic status.

**Variable**		**N**	**% valid**
**Gender**	Male	1,034	44.8
	Female	1,276	55.2
	Missing#	1	

**Age**	17–19 years	69	3.0
	20–29 years	240	10.5
	30–39 years	293	12.8
	40–49 years	420	18.4
	50–59 years	344	15.1
	60–69 years	440	19.3
	70–79 years	322	14.1
	80 years and more	155	6.8
	Missing	28	

**Socio-economic status**	Low	318	14.6
	Middle	1,145	52.5
	High	717	32.9
	Missing	131	

# In one case during the study (between study part I and II) a sex change occurred.

**Table 3.** Number of participants by indicators of aircraft noise exposure.

**Sound level class (L_Aeq_) in dB**	**Day-Evening-Night**		**Day-Night**		**Day**		**Night**	

	**L_den_ in dB**		**L_dn_ in dB**		**L_Aeq,16h_ in dB**		**L_Aeq,8h_ in dB**	

	**N**	**%**	**N**	**%**	**N**	**%**	**N**	**%**
<40	0	4.2	132	5.7	0	0	381	16.5
40–45	98	22.7	560	24.2	363	15.7	741	32.1
45–50	524	26.6	597	25.8	565	24.4	462	20.0
50–55	615	19.2	506	21.9	497	21.5	523	22.6
55–60	443	27.3	516	22.3	700	30.3	204	8.8
≥60	631				186	8.0	0	0.0

Total	2,311	100.0	2,311	100.0	2311	100.0	2,311	100.0

Mean	54.7		54.1		51.9		45.9	
Standard deviation	6.1		5.9		6.2		6.6	
Minimum	42.4		41.9		40.8		24.4	
Maximum	65.9		64.8		62.7		57.6	

**Table 4.** Product-moment correlation between aircraft noise annoyance 12 months before the interview and parameters of aircraft noise exposure.

	**Scale**	**n**	**Equivalent sound level (unweighted, weighted)**			**Mean maximum sound level**		**Number of events above threshold**	
			**L_Aeq,24h_**	**L_den_**	**L_dn_**	**L_max55,24h_**	**L_max70,24h_**	**N_55,24h_**	**N_70,24h_**
Aircraft noise annoyance	5-pt.	2,308	0.45	0.43	0.42	0.39	0.34	0.33	0.34
	11-pt.	2,272	0.43	0.42	0.41	0.36	0.29	0.34	0.34

**Table 5.** Correlations and partial correlations of aircraft sound level (L_den_) and aircraft noise annoyance with selected questionnaire variables.

**Variables**	**Correlation**		**Partial correlation**		

	**Noise annoyance (11 pt.)**	**Noise level L_den_**	**Noise annoyance (11 pt.)^1^**	**Noise level L_den_^2^**	**between annoyance (11 pt.) and L_den_^3^**
*Aircraft noise annoyance*					
annoyance (5-pt.)	0.87	0.43	0.84	0.14	
annoyance (11-pt.)	1.00	0.43	1.00		

*Disturbances of...*					
communication indoor	0.79	0.48	0.74	0.25	0.09
relaxation/concentration indoor	0.79	0.42	0.75	0.15	0.17
communication outdoor	0.81	0.40	0.77	0.11	0.19
relaxation outdoor	0.79	0.38	0.75	0.08	0.22
nocturnal sleep	0.76	0.37	0.72	0.08	0.24

*Coping*					
Measures to cope with noise	0.81	0.41	0.77	0.13	0.17

*Source-related attitudes*					
Negative expections	0.74	0.24	0.72	−0.12	0.38
Positive expections	−0.14	0.01#	−0.16	0.08	0.43
Econom. expectations	−0.40	−0.19	−0.36	−0.02#	0.39
Aircraft-related fears	0.71	0.28	0.68	−0.03#	0.33
Confidence in authorities	−0.35	−0.20	−0.29	−0.06	0.39

*Residential satisfaction*					
Satisfaction with dwelling	−0.04#	−0.12	0.01#	−0.11	0.42
Satisfaction with residential area	−0.28	−0.19	−0.23	−0.08	0.40
Infrastructure	−0.11	0.01#	−0.13	0.08	0.43
Quietness, insulation	−0.47	−0.30	−0.40	−0.21	0.34
Attractiveness, neighbours	−0.17	−0.10	−0.15	−0.02#	0.42
Residential satisfaction (total score)	−0.29	−0.15	−0.26	−0.01#	0.41

*Sensitivity*					
Noise sensitivity	0.36	0.08	0.36	−0.09	0.43

Partial correlation adjusted for

1 L_den_,

2 aircraft noise annoyance (11-pt. scale),

3 variable in row;

# not significant (p > 0.01); n = 2,127–2,311.

**Table 6.** Description of health variables grouped by aircraft sound level at daytime (L_Aeq,16h_) and night-time (L_Aeq,8h_).

**Health variables**	**Aircraft sound level**											
	**at daytime–––L_Aeq,16h_ [dBA]**						**at night-time–––L_Aeq,8h_ [dBA]**					
	**40–45**	**45–50**	**50–55**	**55–60**	**≥60**		**<40**	**40–45**	**45–50**	**50–55**	**≥55**	
*SF12/36 HQoL scores: mean (SD)*												
Vitality (SF36)	70.8 (18.7)	65.9 (17.8)	66.6 (18.7)	67.5 (19.1)	67.8 (17.8)	**	68.3 (18.5)	66.7 (18)	67.9 (18.2)	67.7 (20.1)	67.5 (17.8)	

Mental health (SF36)	77.3 (13.8)	75.6 (14)	73.5 (15.9)	75.5 (15.1)	78.3 (13.7)	**	75.1 (14)	76.0 (14.4)	75.4 (15.6)	75.0 (15.4)	77.1 (13.7)	

Mental health (SF12)	54.1 (6.1)	53.4 (6.9)	52.4 (7.8)	53.6 (6.9)	54.5 (6.6)	**	53.4 (6.3)	53.4 (7.1)	53.2 (7.2)	53.4 (7.2)	54.4 (6.7)	

Physical health (SF12)	51.1 (8.7)	49.5 (9.9)	50.1 (9.2)	49.9 (9.2)	50.1 (9.7)		50.2 (9.5)	49.8 (9.7)	50.6 (8.7)	49.8 (9.4)	49.9 (9.5)	

*GSCL-24 health complaints: mean (SD)*												
Exhaustion	46.1 (9.1)	47.6 (9.9)	48.0 (9.3)	47.7 (9.8)	46.5 (8.6)		47.4 (9.7)	47.8 (9.6)	46.6 (9.3)	47.7 (9.8)	46.9 (8.8)	
Stomach complaints	48.1 (7.4)	48.5 (7.6)	48.6 (8.1)	49.1 (7.8)	46.8 (6.3)	*	49.2 (8.0)	48.6 (7.7)	47.6 (7.1)	49.2 (8.0)	47.1 (6.7)	**
Limb complaints	45.9 (9.3)	47.8 (9.7)	47.1 (9.9)	47.5 (9.7)	44.3 (9.3)	**	47.3 (10.0)	47.3 (9.6)	45.8 (9.6)	48.0 (9.9)	45.4 (9.2)	**
Cardiac complaints	47.6 (7.4)	47.8 (7.6)	48.4 (8.0)	48.4 (8.1)	46.7 (6.9)		48.0 (7.8)	48.2 (7.7)	47.4 (7.6)	48.5 (8.2)	47.2 (7.0)	
Total score	45.5 (9.2)	47.0 (9.5)	47.0 (9.7)	47.2 (9.8)	44.3 (9.0)	**	46.9 (9.7)	47.0 (9.5)	45.4 (9.5)	47.5 (9.8)	45.1 (9.1)	**

*Sleep quality: mean (SD)*												
Sleep quality (PSQI)	3.4 (2.8)	3.8 (3.0)	4.0 (3.1)	4.1 (3.1)	3.4 (2.8)	**	3.7 (2.9)	3.9 (3.1)	3.7 (3.0)	4.2 (3.1)	3.6 (2.8)	*

** p < 0.01;

* p < 0.05 (adjusted for number of tests)

**Table 7.** Description of health variables grouped by aircraft noise exposure and noise sensitivity.

**Health variables**	**Aircraft noise annoyance**						**Noise sensitivity**					
	**not at all**	**slightly**	**moderately**	**very**	**extremely**		**not**	**a little**	**moderately**	**rather**	**very**	
*SF12/36 HQoL scores: mean (SD)*												
Vitality (SF36)	73.6 (18.4)	70.9 (18.0)	68.4 (17.4)	64.6 (17.9)	60.7 (19.0)	**	73.8 (18.0)	69.5 (17.0)	66.9 (17.5)	63.4 (20.4)	53.3 (22.4)	**
Mental health (SF36)	79.6 (13.2)	77.8 (13.8)	76 (14.1)	74.0 (15.0)	71.1 (16.1)	**	81.3 (14.6)	77.6 (13.1)	74.9 (13.8)	71.7 (16.1)	63.9 (18.2)	**
Mental health (SF12)	55.2 (5.1)	54.2 (6.0)	53.5 (6.5)	53.1 (7.2)	51.5 (8.9)	**	55.1 (6.2)	54.3 (5.8)	53.2 (6.8)	51.7 (8.8)	49.4 (9.0)	**
Physical health (SF12)	51.1 (9.3)	51.4 (8.1)	50.2 (8.8)	49.1 (9.7)	48.5 (10.8)	**	51.2 (9.2)	50.9 (8.9)	50.1 (8.8)	48.2 (9.9)	45.3 (12.5)	**

*GSCL-24 health complaints: mean (SD)*												
Exhaustion	44.8 (8.1)	45.1 (7.8)	46.4 (8.7)	48.7 (10.3)	51.7 (10.7)	**	45.0 (8.1)	45.8 (8.2)	47.9 (9.7)	50.0 (10.7)	54.1 (12.1)	**
Stomach complaints	47.5 (7.0)	47.3 (6.6)	48.5 (7.5)	49.1 (8.2)	50.1 (8.4)	**	46.0 (6.3)	48.0 (7.2)	49.1 (7.7)	49.5 (8.4)	51.7 (9.3)	**
Limb complaints	45.7 (8.6)	45.2 (8.2)	46.3 (9.4)	47.9 (10.4)	49.9 (10.9)	**	44.4 (8.9)	45.7 (8.5)	47.5 (9.8)	49.2 (10.6)	52.6 (12.3)	**
Cardiac complaints	46.3 (6.1)	46.5 (6.3)	47.8 (7.6)	49.1 (8.6)	50.1 (8.8)	**	46.1 (6.5)	47.0 (6.7)	48.1 (8)	50.2 (8.9)	52.7 (9.4)	**
Total score	44.4 (8.4)	44.4 (7.9)	46.0 (9.2)	47.8 (10.4)	50.2 (10.5)	**	43.4 (8.4)	45.2 (8.5)	47.3 (9.6)	49.2 (10.5)	52.9 (11.6)	**

*Sleep quality: mean (SD)*												
Sleep quality (PSQI)	2.6 (2.2)	3.2 (2.7)	3.7 (2.7)	4.2 (3.0)	5.5 (3.6)	**	2.7 (2.6)	3.2 (2.6)	4.1 (3.0)	5.2 (3.3)	6.0 (3.6)	**

** p < 0.01;

* p < 0.05 (adjusted for number of tests)

**Table 8.** Associations between aircraft noise exposure at daytime (L_Aeq,16h_), aircraft noise annoyance, noise sensitivity, and health variables (Odds ratios [OR] per unit and ±95% confidence interval [CI]).

Health variables	Aircraft sound level L_Aeq,16h/8h_#			Aircraft noise annoyance			Noise sensitivity		

	OR	CI−	CR+	OR	CI−	CR+	OR	CI−	CR+
*Health-related quality of life (SF36/12 scores < median)*									

Vitality (SF36)	**0.95**	0.93	0.97	**1.25**	1.13	1.37	**1.13**	1.02	1.26
Mental health (SF36)	**0.96**	0.94	0.98	**1.13**	1.03	1.24	**1.40**	1.26	1.55
Mental health (SF12)	**0.96**	0.94	0.98	1.06	0.97	1.17	**1.22**	1.10	1.36
Physical health (SF12)	**0.97**	0.95	0.99	**1.13**	1.01	1.26	**1.19**	1.06	1.34

*GSCL-24 health complaints (above 50% = average of population in Germany)*									
Exhaustion	**0.98**	0.96	1.00	**1.36**	1.23	1.51	**1.40**	1.26	1.56
Stomach complaints	0.99	0.97	1.01	**1.12**	1.02	1.24	**1.18**	1.06	1.30
Limb complaints	**0.96**	0.94	0.98	**1.22**	1.10	1.34	**1.48**	1.33	1.65
Cardiac complaints	**0.96**	0.94	0.98	**1.32**	1.19	1.47	**1.35**	1.21	1.50
Total score	**0.96**	0.94	0.98	**1.41**	1.27	1.56	**1.53**	1.37	1.71

*Sleep quality (bad sleep quality: PSQI score > 5))*									
Bad sleep quality	**0.95**	0.93	0.97	**1.45**	1.29	1.63	**1.42**	1.25	1.61

Adjusted for railway and road traffic sound level, age, gender, socio-economical status, home ownership, residential satisfaction, usual window position in the sleeping room at night, number of hours away from home;

# L_Aeq,8h_ (10 *pm*–6 *am*) for sleep quality, L_Aeq,16h_ (6 *am*–10 *pm*) for all other health variables; bold: OR significant on significance level p < 0.05.

**Table 9.** Results of multi-factorial GLM with HQoL variables as criteria.

**Effect^1^**	**df factor**	**SF 36 vitality**		**SF36 mental health**		**SF12 mental health**		**SF12 physical health**		**GSCL total health complaints**		**PSQI sleep quality**	

		F	p	F	p	F	p	F	p	F	p	F	p
L_Aeq_^2^	4	2.1	0.079	2.8	0.025	4.2	0.002	0.9	0.455	3.9	0.003	1.4	0.223
Morbidity	1	298.2	0.000	83.5	0.000	29.2	0.000	635.6	0.000	314.2	0.000	273	0.000
L_Aeq_ × morbidity	4	4.6	0.001	3.8	0.004	1.8	0.125	3.9	0.004	1.4	0.217	0.5	0.713

df error		1,882		1,882		1,857		1,857		1,844		1,764	

1 Results based on GLM with L_Aeq_ (five 5-dB-classes), morbidity (0–1 *vs.* ≥2 diseases), expectations about residential future (worse *vs.* better/no change), noise sensitivity (median split: low *vs.* high), daytime window position (closed *vs.* open/tilted);

2 PSQI sleep quality: L_Aeq,8h_ for night-time; all others: L_Aeq,16h_ for daytime.

## Appendix

**Appendix 1.** Variables assessed in the questionnaire.

**Variable category**	**Variable**	**Number of items**	**Response scale**	**Cronbach’s *α***	**Ref.**
Annoyance	Aircraft noise annoyance in the last 12 months before the interview	2	intensity scales: verbal 5-pt., numerical 11-pt.		34
Disturbances of activities due to aircraft noise	…of communication indoor	3	5-pt. intensity scale; mean score	0.92	
	…of communication outdoor	1			
	…of relaxation/concentration indoor	2		0.93	
	…relaxation outdoor	1			
	…nocturnal sleep	3		0.92	
Coping with aircraft noise	Measures done within an aircraft noise situation (coping)	16	5-pt. frequency scale; mean score	0.94	
Air traffic related attitudes	Fears concerning air traffic	4	5-pt. intensity scale; mean score	0.86	
	Confidence in authorities’ effort for aircraft noise reduction	7		0.86	
	*Expectation concerning airport expansion*				
	Negative expectation	6	5-pt. intensity scale; mean score	0.91	
	Positive expectation	3		0.71	
	Economic expectation	2		0.76	
Residential satisfaction	Satisfaction with dwelling	1	5-pt. intensity scale		
	Satisfaction with residential area	1	5-pt. intensity scale		
	Satisfaction with infrastructure (6 items), attractiveness of residential area (3 items), quietness (3 items)	12	5-pt. intensity scale; subscores and total score: mean scores	0.82	32
Health-related quality of life (HQoL)	Vitality (SF-36)	4	SF subscales: Transformed scale with values between 0 and 100. Higher values indicate higher HQoL		35
	Mental health (SF-36)	5			35
	Mental health (SF-12)	6			35
	Physical health (SF-12)	6			35
	Health complaints (GSCL 24): exhaustion (6 items); stomach (6), limbs (6); cardiac (6), total (24)	24	Subscores transformed to scale from 0 to 100. Reference sample [36]: mean = 50, SD = 10		36
Health diseases (morbidity)	Self-reported diagnosed diseases; Multimorbidity: 0–1 diseases *vs.* 2 or more diseases	18	ever had; in the last 12 months; dichotomized		37;38
Sleep	Sleep quality (PSQI total index)	18	sum score: 0 to 21; values > 5 = bad quality		39
Personal factors	Noise sensitivity (single item)	1	5-pt. intensity scale		
	Age				
	Gender		female / male		
	House ownership		tenant / owner		
	Socio-economical status ‘Scheuch-Winkler index’	3	includes income, education, occupational status		40
